# 2211. Nursing Home Infection Preventionists are Leading Antimicrobial Stewardship Programs – Should They Be? A Statewide Survey of Stewardship Leadership Roles in Nursing Homes

**DOI:** 10.1093/ofid/ofad500.1833

**Published:** 2023-11-27

**Authors:** Jessica Zering, Katarina Kamenar, Marisa A D’Angeli, Samantha Delmer

**Affiliations:** Washington State Department of Health, Richland, Washington; Washington State Department of Health, Richland, Washington; Washington State Department of Health, Richland, Washington; Washington State Department of Health, Richland, Washington

## Abstract

**Background:**

The Centers for Disease Control’s Core Elements (CEs) of Antibiotic Stewardship for Nursing Homes is the best-practice guideline for developing a nursing home (NH) antibiotic stewardship program (ASP). The CEs recommend that NHs select a medical director, director of nursing, and/or consultant pharmacist to lead the ASP. The Centers for Medicare and Medicaid Services’ State Operations Manual currently suggests an ASP leader and defines pharmacist involvement in ASPs as the performance of a medication regimen review at least once monthly. A survey was conducted of NHs in Washington State to assess implementation of ASP leadership best practices.

**Methods:**

Infection Preventionists (IP) in licensed NHs in Washington State were contacted via email and asked to complete an online REDCap survey on their ASP. Non-responder facilities were contacted via phone and asked that the employee most knowledgeable about their ASP complete the survey. Responses were collected between February 15th, 2022, to May 2, 2023.

**Results:**

A total of 101 Washington NHs out of 198 contacted (51%) responded to the survey. Approximately 90% of respondents reported that IPs led or co-led their antibiotic stewardship program (Figure 1). In comparison, directors of nursing were identified as leaders or co-leaders by 50% of respondents, medical directors by 37%, and consultant pharmacists by 34%. Subgroup analyses by number of facility licensed beds, corporate association, and hospital affiliation demonstrated that IPs were the staff primarily responsible for antibiotic stewardship efforts in NHs across Washington State (Figure 2). Common barriers reported to identifying an ASP lead were competing demands, lack of prioritization by leadership, staffing shortages and lack of expertise.

**Figure:**
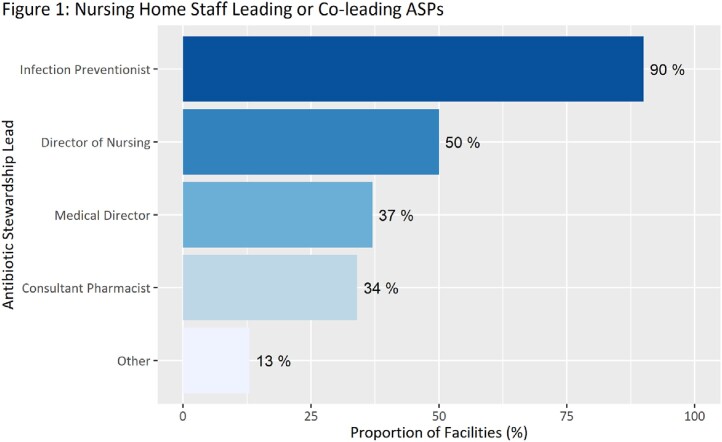

**Figure:**
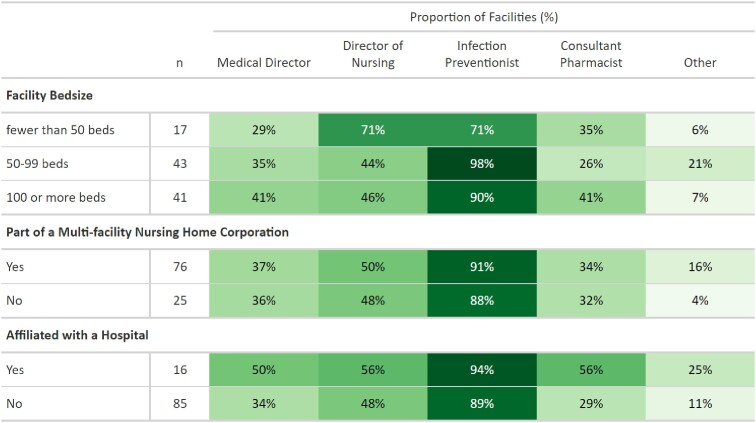

**Conclusion:**

There is a discrepancy between NH ASP best practice recommendations and on-the-ground implementation. NH administrators can ensure alignment with best practices by putting dedicated time and responsibilities for stewardship into the job descriptions of medical directors and directors of nursing. Regulators can promote pharmacy leadership of NH ASPs by requiring greater clinical expertise and involvement of pharmacists beyond once-monthly medication regimen reviews.

**Disclosures:**

**All Authors**: No reported disclosures

